# The costs and effects of a nationwide insecticide-treated net programme: the case of Malawi

**DOI:** 10.1186/1475-2875-4-22

**Published:** 2005-05-10

**Authors:** Warren Stevens, Virginia Wiseman, Juan Ortiz, Desmond Chavasse

**Affiliations:** 1 MRC Laboratories, PO Box 273, Banjul, The Gambia; 2 Health Policy Unit, London School of Hygiene & Tropical Medicine, Keppel Street, London, WC1E 7HT, UK; 3 UNICEF, P.O. Box 30375. Lilongwe 3, Malawi; 4 PSI Malaria Department, Po Box 22591-0400, Nairobi, Kenya

## Abstract

**Background:**

Insecticide-treated nets (ITNs) are a proven intervention to reduce the burden of malaria, yet there remains a debate as to the best method of ensuring they are universally utilized. This study is a cost-effectiveness analysis of an intervention in Malawi that started in 1998, in Blantyre district, before expanding nationwide. Over the 5-year period, 1.5 million ITNs were sold.

**Methods:**

The costs were calculated retrospectively through analysis of expenditure data. Costs and effects were measured as cost per treated-net year (cost/TNY) and cost per net distributed.

**Results:**

The mean cost/TNY was calculated at $4.41, and the mean cost/ITN distributed at $2.63. It also shows evidence of economies of scale, with the cost/TNY falling from $7.69 in year one (72,196 ITN) to $3.44 in year five (720,577 ITN). Cost/ITN distributed dropped from $5.04 to $1.92.

**Conclusion:**

Combining targeting and social marketing has the potential of being both cost-effective and capable of achieving high levels of coverage, and it is possible that increasing returns to scale can be achieved.

## Background

The cost-effectiveness of insecticide-treated nets in reducing morbidity and mortality in malaria endemic countries has been proven time and again [1-3]. I It is considered to be one of the most cost-effective ways of reducing the burden of malaria, with an estimated cost per Disability Adjusted Life Year (DALY) averted of under $50 [4]. However most studies have been undertaken along side trials, and as such they equate to little more than measures of cost-efficacy, leaving a need for better estimates of the true cost-effectiveness of such programmes in practice. With one of the main Roll Back Malaria goals being to achieve at least 60% coverage of pregnant women and children under five years of age with ITNs, evaluations of the cost-effectiveness of different methods of financing and delivering nets on a large scale is desperately needed.

Differences in measures of cost-effectiveness in trials and in interventions in practice are understandable, due to the inevitable changes in returns to scale associated with the scaling up of interventions [5,6], the operational difficulties associated with programmes of this size [7,8] and the constant returns to scale normally associated with primary health care delivery systems. [9,10] A number of interventions, that have been evaluated alongside trials, have been shown to be far less effective and often more costly, once they have been scaled up and undertaken outside the confines of the trial setting [11].

The debate as to the best way to achieve long-term shifts in levels of ITN utilization in malaria endemic countries has centered around the trade-off between the need for immediate health impact and the need for long-term sustainability of such a change in coverage. Those who advocate the universal distribution of free nets have prioritized the need for immediate results in terms of health gain, whereas those who argue for the development of domestic markets for ITNs wish to ensure the long-term sustainability of utilization of ITNs. The Malawi model is a third way that combines traditional, social marketing with heavily subsidized highly-targeted distribution through the nationwide network of public health facilities. Social marketing has been defined as the application of 'commercial marketing technologies to the analysis, planning, execution, and evaluation of programmes designed to influence the voluntary behaviour of target audiences in order to improve their personal welfare and that of their society' [12]. In addition to this, the model has presented itself with the challenge of achieving high coverage levels in a relatively short period of time, while developing and sustaining a local market for ITNs which could sustain itself into the future, when donor money reprioritizes or dries up.

While there is a small but growing literature looking at the application of social marketing techniques to ITN distribution [2,13-17] the same cannot be said for economic analyses. It has been said that social marketing has additional costs that free distribution nets do not, such as advertising, branding, promotion and retailers' margins, yet there has been only one published, cost-effectiveness study of a social marketing project and that was deemed to be cost-effective [2]. Currently the literature on the cost-effectiveness of ITN distribution interventions is measured using only the immediate, directly relevant health outcomes, and ignores any benefits from developing the market for future accessibility. This is understandable, as conventional forms of economic evaluation tend to overlook issues of sustainability [18]. Nevertheless its value comes in practicality; in the ability to make comparisons between different methodologies with broadly similar goals, and most fundamentally, in recognizing that the resources available for health services are insufficient to meet all the potential uses for them. This evaluation looks at the cost-effectiveness of a specific social marketing ITN intervention in terms of cost per ITN distributed and cost per treated-net-year.

In 1998, USAID contracted Population Services International/ Malawi to design and implement a social marketing ITN programme in Blantyre district. The programme strategy adopted has been published in detail elsewhere [19]. In brief, the strategy involves segmenting the market such that a more expensive blue conical net (with insecticide treatment kit) is made available to consumers for $5–6 through private sector outlets, targeting those who can afford a commercially priced net. A subsidized green rectangular net (with a kit) is made available to pregnant women and children under 5 for $0.6, through public health facilities. The nets were branded and heavily promoted to the public through a range of mass media and interpersonal communications channels.

Over the next three years the delivery of both the commercially available net and the health facility model was expanded nationwide, thanks to a collaborative effort involving the Malawi Government, UNICEF, USAID and DFID. By January 2003, ITNs were being delivered through commercial outlets and public health facilities in all 27 districts of the country. To further improve access to ITNs, in 2003, unbranded green rectangular nets (with a kit) were delivered via community-based groups at the subsidised price of $1.2. During the latter 12 month period reported here (October 2002 – September 2003), a total of 942,000 nets were sold of which 8% were blue conical nets, 16% were unbranded green rectangular nets delivered via community-based channels and 76% were green rectangular nets delivered through public health facilities. At the time of writing, the programme continues to sell about 100,000 ITNs per month nationwide with roughly the same proportion of each type of net.

## Methods

The costs were taken from financial expenditure data collected over the course of the first five years of the programme. As the study was retrospective, no research costs are included. The costs relate to Blantyre District only in year one, and nationwide from year two.

Costs were broken down into capital costs, which were annualized and discounted across their life span, and recurrent or programme costs. The latter were broken down into direct costs associated with the ITN programme, and shared costs which were apportioned using one of three indicators. The first was budget headings, the second was total volume of product and the third was total sales calls by agents. An appropriate apportionment method was chosen for each type of expenditure, for example, in terms of apportioning warehouse rental, product volume was used, as ITNs take up much more space than condoms or sachets of oral rehydration salts, which are also delivered by PSI/Malawi. Whereas, with cost of sales commissions, the number of sales calls was used, which better represented the relative effort of the sales agents.

Capital items were annualized over assumed life spans, taken directly from the KINET study ^2 ^so that comparison with other studies, the main aim of this study, could be more transparent. The brand was estimated at seven years; billboards and vehicles were eight years, and computers, furniture and the bed-nets themselves were five years. The discount rate used was 3%, and costs were measured in a combination of local currency (Malawi Kwacha) and in US dollars, depending on whether the resources were purchased or paid locally or overseas. All costs were then translated into US dollars on the MK-US$ exchange rate for July 1^st ^of that year.

## Results

### Costs

The financial cost of the programme, shown in Table 1, over the first five years was just over $6 million, with an economic cost from 1998–2003 of just over $3,500,000. In this time, a little under 1.5 million ITNs and 300,000 re-treatment kits were sold. Table 2 shows the breakdown of the costs by line item, with set-up costs making up 3%, capital costs making up 57% and recurrent costs making up 40%. The biggest cost was that of the nets and the insecticide, which made up 60% of total costs, followed by staff, which made up 10%. Overhead costs, including local overheads, and the cost of technical support from the PSI/Washington office was also 10%.

**Table 1:** Financial and economic costs of the programme from 1999–2003 (1999 prices).

	Total financial cost (US $)	Total economic cost (US $)
Brand creation / market research	**146,801**	**107,085**
		
Capital costs		
Vehicles	157,552	50,228
Equipment & furniture	15,468	15,468
ITNs	4,478,365	2,147,400
**Subtotal**	**4,651,385**	**2,213,096**
		

		
Recurrent costs		
Insecticide	191,555	191,555
Staff	357,204	357,204
Fuel/maintenance	339,346	339,346
Office /warehouse rental	45,672	45,672
Advertising & Promotion	272,646	272,646
Supplies/overheads	351,682	351,682
Subtotal	**1,558,104**	**1,558,104**
		
Total cost	**6,356,290**	**3, 878,287**
		

		
*ITNS distributed*		*1,471,941*
*Retreatments distributed*		*287,079*
***Treated net years***		***879,510***
		

		
**Cost per net distributed**		**2.63**
**Cost per treated net year**		**4.41**

**Table 2:** Annual costs and cost-effectiveness ratios of the programme 1999–2003 (1999 prices)

	1999	2000	2001	2002	2003	Average	(%)
Brand creation	**20,972**	**21,161**	**21,279**	**21,687**	**21,986**	**21,417**	**3%**
							
Capital costs							
Vehicles	4,056	6,144	8,381	14,595	17,053	10,046	1%
Equipment & furniture	1,903	3,288	4,208	2,848	3,221	3,094	0%
ITNs	77,394	202,893	348,829	586,770	931,515	429,480	55%
**Subtotal**	**83,353**	**212,325**	**361,418**	**604,213**	**951,789**	**442,619**	**57%**
							

Recurrent costs							
Insecticide	16,753	34,335	38,808	58,076	43,582	38,311	4%
Staff	81,496	90,572	54,058	64,104	66,974	71,441	10%
Fuel/maintenance	37,346	67,105	44,115	94,223	96,558	67,869	9%
Office /warehouse rental	806	6,380	10,204	14,202	14,080	9,134	1%
Advertising & Promotion	83,960	71,846	48,149	27,603	41,087	54,529	7%
Supplies/overheads	38,954	42,492	42,905	82,322	145,008	70,336	10%
**Subtotal**	**259,315**	**312,731**	**238,240**	**340,531**	**407,288**	**311,621**	**40%**
							
*ITNS distributed*	72,196	131,881	174,376	372,911	720,577	294,388	
*Retreatments distributed*	22,337	46,731	54,103	81,824	82,084	57,416	
***Treated-net-years***	**47,267**	**89,306**	**114,240**	**227,368**	**401,331**	**175,902**	


Total cost	**363,640**	**546,217**	**620,937**	**966,431**	**1,381,063**	**775,657**	**100%**
Cost per net distributed	5.04	4.14	3.56	2.59	1.92	2.63	
**Cost per treated-net-year**	**7.69**	**6.12**	**5.44**	**4.25**	**3.44**	**4.41**	

### Consequences

As this is a retrospective evaluation of an ongoing working programme, rather than a trial, no health impact data was collected, and so the focus is on process outcomes, including the number of nets distributed and the number of treated net years. The latter measure comes from previous literature on CEA of ITNs [2] and does not require direct translation into health benefits. This allows the results from this study to be compared with other studies. The choice of cost per treated-net-year (TNY) is conservative, as it assumes zero benefit from an untreated net and insecticide treatment is assumed to last 6 months. The discussion draws from the literature on the efficacy of ITNs to estimate the likely health impact and more recognizable measures of cost-effectiveness.

### Cost and effectiveness

The average economic cost per net delivered and the average cost per treated-net-year, over the five years, are $2.63 and $4.41 respectively. This compares favourably to other studies where estimates of $8 and $4 [2,3] have been shown. The interesting aspect of this study is the gains from cost savings from producing at higher levels, or what is often termed scale efficiency savings (SES) that are made, as shown in Table 2, where the cost per treated-net-year drops from $7.69 to $3.44 as throughput rises from 72,196 to 720,577 ITNs.

## Discussion

It is vital for policy-makers to have information on the costs and effects of scaling up malaria interventions, including the provision of ITNs. Recent work in this area has undoubtedly helped to begin addressing this issue. For example, the work by the Commission for Macroeconomics and Health has provided preliminary estimates of costs of scaling up 5 malaria-related interventions including diagnosis and treatment for over-fives, chemoprophylaxis or presumptive treatment for pregnant women, provision of insecticide-treated nets and residual household spraying for malaria prevention [22]. A number of countries are also experimenting with scaling up of interventions designed to improve the home management of malaria. Lessons learnt in Ghana, Uganda, Nigeria, Burkina Faso, Zambia and Kenya are now being shared [23]. While there is no doubt that researchers are paying more attention to issues surrounding the costs and effects of scaling up malaria interventions, there is still a way to go, especially with regard to the delivery of ITNs. In particular, the costs of scaling up ITNs are currently restricted to a relatively small number of studies based on the evaluation of trials or research studies that are often of limited scale. These studies also fail to account for the inevitable growth of scaling up over time that is present in implementation of many public health interventions. The purpose of this study is to start to address these gaps by incorporating ongoing fieldwork into the cost-effectiveness debate around the delivery of ITNs.

### Scaling up of ITN delivery

One of the key findings of this study is that there are considerable scale-efficiency savings to be made. Table 3 shows us the scale efficiency savings (SES) over the five years and relates them to increases in throughput of ITNs. SES has been separated into two components, the 'procurement SES' and the 'distribution SES'. This allows us to see to what extent the SES are a component of this particular method of distribution and what part of the SES is due primarily to the greater bargaining power of scale. As can be seen from this data, approximately half of the relative efficiency savings over time are due to lowering product or procurement costs.

**Table 3:** Breakdown of total scale efficiency savings into 'procurement' and 'distribution' costs

Financial unit costs	**1999**	**2000**	**2001**	**2002**	**2003**	**Totals/Average**
***ITN unit cost***	5.36	4.67	4.01	3.05	2.27	3.04
***Retreatment pack u/c***	0.75	0.75	0.75	0.75	0.57	0.70
						
***ITNs***	72,196	131,881	174,376	372,911	720,577	1,471,941
***Retreatment packs***	22,337	46,731	54,103	81,824	82,084	287,079
***Treated net years***	47,267	89,306	114,240	227,368	401,331	879,510
***Output growth (actual)***		42,040	24,934	113,128	173,963	478,180
**Output growth (%)**		89%	28%	99%	77%	**73%**
						
***Procurement U/C***	5.36	4.67	4.01	3.05	2.27	
***U/C savings***		0.69	0.66	0.96	0.78	***3.09***
**SES**		13%	14%	24%	26%	**58%**
						
***Distribution U/C***	3.73	2.33	1.32	0.85	0.56	
***U/C savings***		1.40	1.01	0.47	0.30	***3.18***
**SES**		37%	43%	36%	35%	**85%**
						
***Total U/C***	9.09	7.00	5.33	3.90	2.83	
***Savings U/C***		2.09	1.67	1.43	1.08	***6.27***
**SES**		23%	24%	27%	28%	**69%**

It could be estimated that these SES could have been enjoyed by any of the alternative methods of distribution, whereas the distribution SES is probably more closely related to the specifics of the distribution method employed, although this is purely speculation, as there is no comparator. What we can say is that health systems, particularly public sector health systems are not renowned for their economies of scale or for falling marginal costs, and that recent studies looking at opening public services up to private competition has tended to increase cost-efficiency and returns to scale [20]. One thing is certain, there is a growing belief that the reliance on an assumption of constant returns to scale is limiting the value and practicality of cost-effectiveness studies to policy makers [5,21].

Currently, despite the evidence of the relative cost-effectiveness of ITNs and the goals and the political commitment of the RBM partners to increase coverage throughout sub-Saharan Africa beyond the 60% mark, there has not been the speed of progress in scaling up of this intervention that has been required. A recent study by the CDC and UNICEF looked at changes in ITN and bed-net usage in Malawi as a whole between 2000 and 2003 [22]. Households of at least one net have risen from 12% to 43%. In target groups, 35% of children under 5 slept under a net the previous night, up from 8% and similarly, 32% of pregnant women slept under a net the previous night, up from just 8% three years previously. These are significant changes in a relatively short period of time.

At this time, this is partly due to a lack of financial commitment which is, in turn, due to a lack of a consensus on: a) how best to undertake this scale up; and b) how much it is likely to cost. The first of these two cannot be answered unless the desired output, be it pure short-term health gain, long-term development of a sustainable net culture, or both, is clarified. The second needs to consider the fact that economies-of-scale may exist. If estimates of the cost of achieving certain targets are based on cost-effectiveness data from small scale trials, and modeled at constant returns to scale there is a chance this could drastically overestimate the true cost, if the evidence presented here on returns to scale is not an anomaly. Without more evaluations of programmes at scale to compare, this can only be speculation.

## Conclusion

The efficacy of insecticide-treated nets for reducing the burden of malaria in sub-Saharan Africa has been repeatedly proven and the debate in this field should, and to some extent has, moved beyond marginal value to one of measuring marginal productivity. The goal for economists and policymakers is to determine the method, or combinations of methods, that can ensure the best way of achieving a sustainable high-level of utilization of this product in communities where the benefits are highest.

It is more than likely that with the dual goals of health impact through high coverage levels, and long-term sustainability of the supply of ITNs in those same countries, that a strategy which involves a combination of different methods of distribution will be required. This paper suggests that a combination of standard social marketing techniques, combined with targeting vulnerable groups with highly subsidized ITNs through both the commercial and formal health care sectors, could achieve relatively high-levels of coverage in both urban and rural areas and in vulnerable groups over time with proper investment.

In addition, contrary to the weight of evidence on scaling up of public health interventions, and of primary health care in general, it may be possible to achieve these high levels of ITN distribution and rapid increases in coverage while keeping unit costs down and achieving increasing returns to scale. To justify such a conclusion, and to compare and contrast with other hybrid methods of ITN delivery, economic evaluations of large-scale programmes need to be carried out.

## Authors' contributions

Warren Stevens compiled and analyzed the data and wrote the first draft of the paper. Virginia Wiseman advised on methodology and analysis and was involved in redrafting of the paper. Juan Ortiz and Desmond Chavasse conceived of the study, were involved in data collection and also in the development of the intervention, and redrafting of the final paper.
